# Computational analysis of prodomain cysteines in human TGF-β proteins reveals frequent loss of disulfide-dependent regulation in tumors

**DOI:** 10.1093/g3journal/jkac271

**Published:** 2022-10-10

**Authors:** Samantha M Daly, Ashley Peraza, Stuart J Newfeld

**Affiliations:** School of Life Sciences, Arizona State University, Tempe, AZ 85287-4501, USA; School of Life Sciences, Arizona State University, Tempe, AZ 85287-4501, USA; School of Life Sciences, Arizona State University, Tempe, AZ 85287-4501, USA

**Keywords:** adenocarcinoma, LTBP, Fibrillin, Genome Data Commons

## Abstract

The functionally diverse members of the human Transforming Growth Factor-β (TGF-β) family are tightly regulated. TGF-β regulation includes 2 disulfide-dependent mechanisms—dimerization and partner protein binding. The specific cysteines participating in these regulatory mechanisms are known in just 3 of the 33 human TGF-β proteins. Human prodomain alignments revealed that 24 TGF-β prodomains contain conserved cysteines in 2 highly exposed locations. There are 3 in the region of the β8 helix that mediates dimerization near the prodomain carboxy terminus. There are 2 in the Association region that mediates partner protein binding near the prodomain amino terminus. The alignments predict the specific cysteines contributing to disulfide-dependent regulation of 72% of human TGF-β proteins. Database mining then identified 9 conserved prodomain cysteine mutations and their disease phenotypes in 7 TGF-β proteins. Three common adenoma phenotypes for prodomain cysteine mutations suggested 7 new regulatory heterodimer pairs. Two common adenoma phenotypes for prodomain and binding partner cysteine mutations revealed 17 new regulatory interactions. Overall, the analysis of human TGF-β prodomains suggests a significantly expanded scope of disulfide-dependent regulation by heterodimerization and partner protein binding; regulation that is often lost in tumors.

## Introduction

Secreted signaling proteins in the Transforming Growth Factor-β (TGF-β) family modulate a vast array of cellular processes in metazoan animals from sponges to humans (Hinck *et al.* 2016). In humans, developmentally defective TGF-β signaling, either loss or gain of function causes birth defects such as hereditary hemorrhagic telangiectasia or fibrodysplasia ossificans progressiva (Haupt *et al*. 2019; Balachandar *et al.* 2022). Later in life, gain of TGF-β signaling facilitates fibrosis (Meng *et al.* 2016) while the loss of TGF-β anti-mitotic signals leads to tumors (Derynck *et al*. 2021). The severe mutant phenotypes produced by flawed TGF-β signaling dictate that these proteins be tightly regulated. We employ a computational approach exploiting amino acid conservation and common mutant phenotypes to suggest new tumor-associated regulatory interactions in the TGF-β family.

One mechanism of regulation is intrinsic to TGF-β proteins. All family members are encoded as 3 domains. At the amino terminus there is a signal sequence that is removed prior to secretion. Downstream is a roughly 250 residue prodomain that is also cleaved prior to secretion. Unlike the signal sequence, after cleavage, the prodomain remains associated with the carboxy-terminal ligand domain. The ligand domain is roughly 110 residues and contains a stereotypical pattern of 6–9 cysteines. Proper folding and dimerization of the ligand domain are facilitated by disulfide bonds between cysteines in the prodomains of 2 TGF-β monomers. Cysteines contributing to dimerization have only been identified in 3 human TGF-β proteins.

An extrinsic mechanism of regulation via binding partners has been shown for TGFB1. Regulation is implemented via disulfide bonds between a single prodomain cysteine in 2 TGFB1 monomers and a pair of cysteines in LTBP1 (Latent TGF-β-Binding Protein; Saharinen and Keski-Oja 2000) or LRC32 (GARP; Wang *et al.* 2012). TGFB1 proteins are secreted as multisubunit latent complexes containing 2 disulfide-linked monomers each with a distinct disulfide bond to LTBP1 (Rifkin *et al.* 2022). Latent complexes are unable to bind receptors and limit the possibility of erroneous TGFB1 signaling. The specific cysteines participating in TGF-β binding have only been identified in these 2 partners out of 10 proteins with the potential to form disulfide bonds with TGF-β family members.

Human TGF-β family members are divided into 3 subfamilies. The TGF-β subfamily has 8 members. We maintain the following naming hierarchy: TGF-β family is all 33 proteins, TGF-β subfamily is 8 proteins, individual proteins such as TGFB1 use their formal name and the group of siblings is TGFB1-3. The Activin subfamily has 8 members. The BMP subfamily has 17 members. Subfamilies are distinguishable by their amino acid sequences (Wisotzkey and Newfeld 2020) and representative homodimers have distinct structures.

TGFB1 has a closed-ring conformation. The prodomain β8 region contains 2 cysteines (Cys223/225) that facilitate homodimerization via an interchain bond between 2 monomers (Shi *et al.* 2011). INHBA in the Activin subfamily (also called ActivinA) has 2 cysteines (Cys244/247) in a prodomain loop between α6 and β8 that form an intrachain bond. INHBA homodimers display a cross-arm conformation (Wang *et al.* 2016). BMP9 (now formally GDF2) has 2 prodomain cysteines (Cys156/237) in a loop between β7 and β9′ that form an intrachain bond. BMP9 homodimers exhibit a widely open conformation (Mi *et al.* 2015).

To better understand the function of TGF-β family prodomains, we previously generated alignments and trees of this domain from mouse, fly, and nematode proteins (Wisotzkey and Newfeld 2020). That data suggested that heterodimers were more common than currently appreciated. Here, we applied the same methods in an analysis of the 33 human TGF-β family members. We identified conserved prodomain cysteines, retrieved mutations in these cysteines from public databases, and utilized common mutant phenotypes to generate hypotheses for disulfide-based regulation via heterodimers or binding partners.

Human prodomain alignments revealed that 24 TGF-β prodomains contain conserved cysteines in 2 highly exposed locations. There are 3 in region of the β8 helix that mediates dimerization near the prodomain carboxy terminus. There are 2 in the Association (Assn) region that mediates partner protein binding near the prodomain amino terminus. The alignments predict the specific cysteines contributing to disulfide-dependent regulation of 72% of human TGF-β proteins. Database mining then identified 9 conserved prodomain cysteine mutations and their disease phenotypes in 7 TGF-β proteins. Three common adenoma phenotypes for prodomain cysteine mutations suggested 7 new regulatory heterodimer pairs. Two common adenoma phenotypes for prodomain and binding partner cysteine mutations revealed 17 new regulatory interactions. Overall, the analysis of human TGF-β prodomains suggests a significantly expanded scope of disulfide-dependent regulation by heterodimerization and partner protein binding; regulation that is often lost in tumors.

## Materials and methods

### Prodomain sequences and alignments

The newest version of the longest isoform of each human TGF-β protein was identified via BLASTp (blast.ncbi.nlm.nih.gov; Gish and States 1993) employing the mouse sequence as a query (Wisotzkey and Newfeld 2020). Human GDNF was retained as a comparator for consistency with previous results. We utilized the UniProt database (www.uniprot.org; Uniprot Consortium 2021) to identify the signal sequence at the amino terminus of the prodomain. The signal sequence was removed from the prodomain sequence before alignment. The mouse cleavage site that separates the prodomain and ligand was employed to identify the human cleavage site at the carboxy terminus of the prodomain. The ligand was removed from the prodomain sequence before alignment. The full set of trimmed human prodomain sequences was aligned with default settings in Clustal Omega (www.ebi.ac.uk/Tools/msa/clustalo; Sievers *et al.* 2020). Employing Clustal allowed us to maintain the continuity of underlying assumptions with our prior analysis of model organism TGF-β prodomains. BoxShade 3.3.1 (ftp://www.isrec.isb-sib.ch/pub/software/unix/boxshade/3.3.1/; written by Kay Hofmann and Michael Baron) was utilized to annotate the alignment employing a cutoff for shading of 20% amino acid similarity and a biochemical definition of similar amino acids (Wisotzkey and Newfeld 2020). This was followed by manual alignment of structural features according to an alignment in Mi *et al.* (2015) to minimize the impact of length variability on the amino and carboxy termini.

### Prodomain and binding partner cysteine mutations and disease phenotypes

For all prodomains and partner proteins, we mined 3 databases of deleterious disease mutations: GeneCards (www.genecards.org; Safran *et al.* 2022), MalaCards (www.malacards.org; Rappaport *et al.* 2017), and the National Cancer Institute Genome Data Commons (portal.GDC.cancer.gov; Grossman *et al.* 2016). Within these databases, manual scanning of each protein’s complete profile led to the capture of every mutation that impacted cysteine. These include: (1) loss-of-function mutations “from a cysteine” to another residue and (2) gain-of-function mutations “to a cysteine” from another residue. We recorded each mutation’s location and their phenotype if any. The majority of phenotypes were from GDC, since many disease phenotypes in the other databases were not connected to a specific residue. Common phenotypes for conserved cysteine mutations in 2 prodomains were identified. All cysteine loss-of-function mutations in each of the 10 potential prodomain-binding proteins were examined for phenotypes matching mutations in TGF-β prodomain conserved cysteines.

## Results

### Identification of conserved cysteines in the prodomain of human TGF-β proteins

**Fig. 1.**
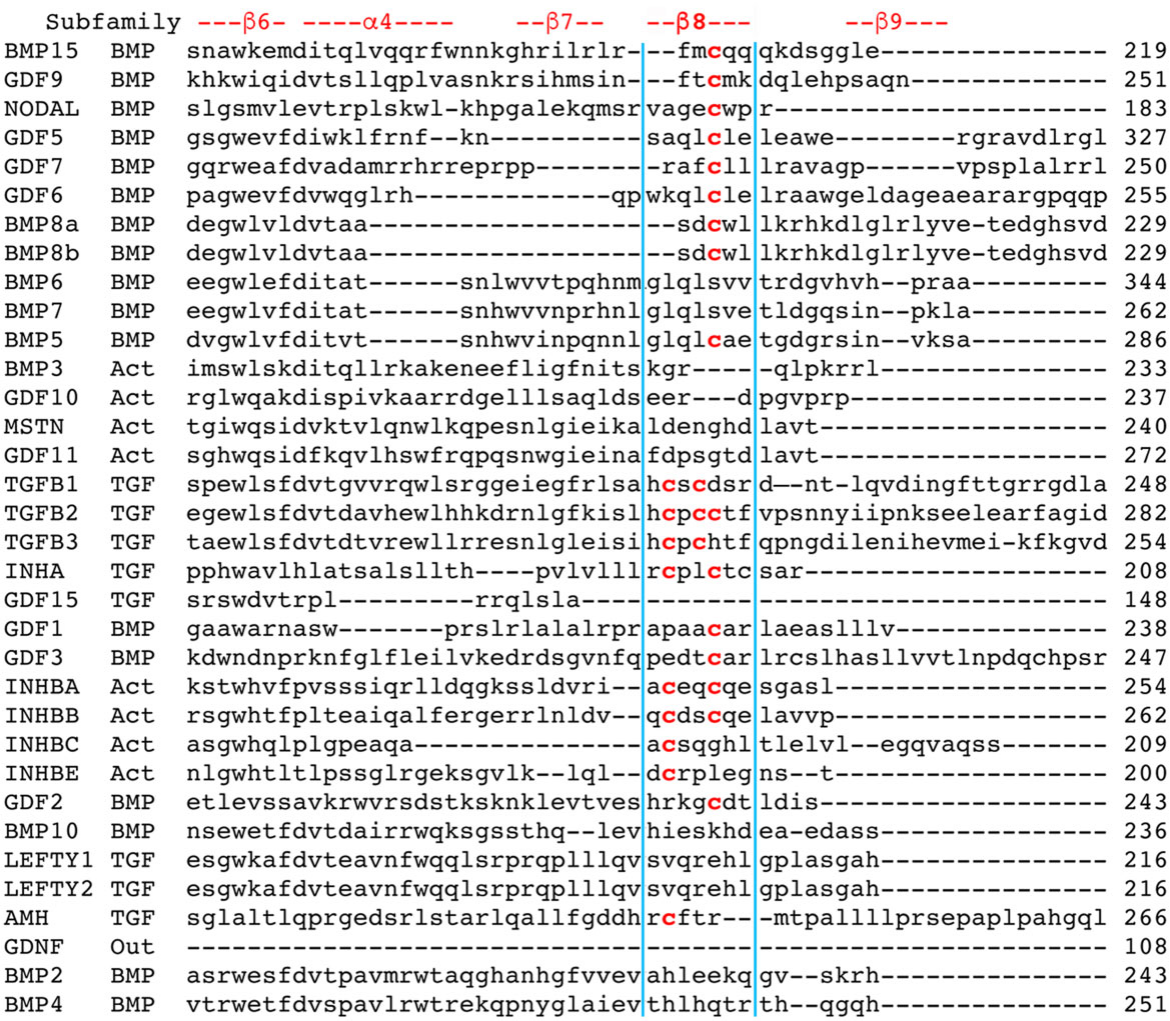
Prodomain β8 region structurally guided refined alignment. Alignment of 60 residues begins upstream of β6 and ends downstream of β9. Noted at the top are structural features derived from the alignment of TGFB1 and BMP9 shown in Mi *et al.* (2015). The β8 helix is further identified by a blue boundary. Note BMP9 is shown here employing its synonym GDF2. Any inconsistency with the carboxy terminus of the prodomain alignments in Wisotzkey and Newfeld (2020) is due to the presence of distinct species and the absence of structural information. Numbering is accurate and indicates the last residue on each line. One, two, or three red cysteines in the β8 region are conserved in 21 of the 33 human TGF-β family proteins. A cysteine conservation summary is in Table 1 and a cysteine mutation summary is in Table 2.

**Fig. 2.**
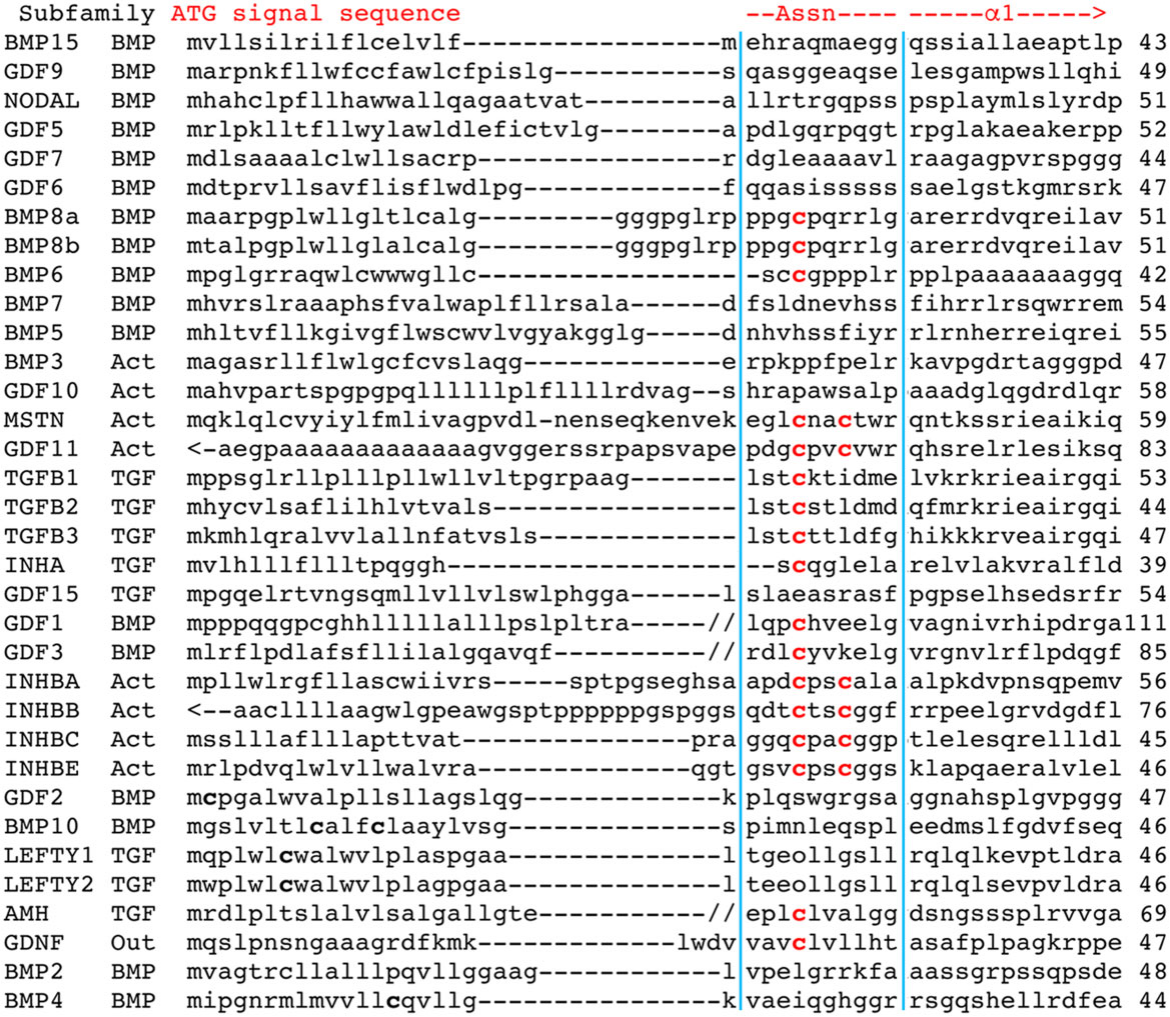
Prodomain Assn region structurally guided refined alignment. Alignment of 60 residues begins at Met1 for all proteins except GDF11 and INHBB due to long sequences between the signal sequence and Assn region. The ATG, signal sequence, and structural features derived from the alignment of TGFB1 and BMP9 (Hinck *et al.* 2016) are noted at the top. The Assn region is further identified by a blue boundary. Note that any inconsistency with the amino terminus of the alignments in Wisotzkey and Newfeld (2020) is due to the presence of distinct species and the absence of structural information. Numbering is accurate and indicates the last residue on each line. One or 2 red cysteines in the Assn region are conserved in 16 of the 33 human TGF-β proteins plus GDNF. A cysteine conservation summary is in Table 1 and a cysteine mutation summary is in Table 2.

Building on the approach of Wisotzkey and Newfeld (2020), we downloaded the longest isoform for each of the 33 human TGF-β sequences plus GDNF (Supplementary Table 1), trimmed each at the signal sequence and ligand cleavage site (Supplementary Table 2), and then created a complete prodomain alignment. To neutralize the impact of length variability at the termini, we implemented structure-based refinements to create alignments of the carboxy and amino termini (Figs. 1 and 2).

The alignments revealed 5 distinct positions that contain conserved prodomain cysteines in 24 human TGF-β family members (Table 1). These include the human homologs of 13 mouse proteins identified previously (Wisotzkey and Newfeld 2020) plus 11 newly identified proteins. To codify specific conserved cysteine positions across proteins, we created a naming convention. Cys@1 is the first conserved cysteine in either the β8 or Assn region. For example, TGFB2 Cys254 is β8Cys@1. Cys@3 is the middle conserved cysteine (third residue in the region). For example, TGFB2 Cys256 is β8Cys@3. Cys@4 is the last conserved cysteine (fourth residue in the region). For example, TGFB2 Cys257 is β8Cys@4.

**Table 1.** Twenty-four human TGF-β proteins with conserved prodomain cysteines.

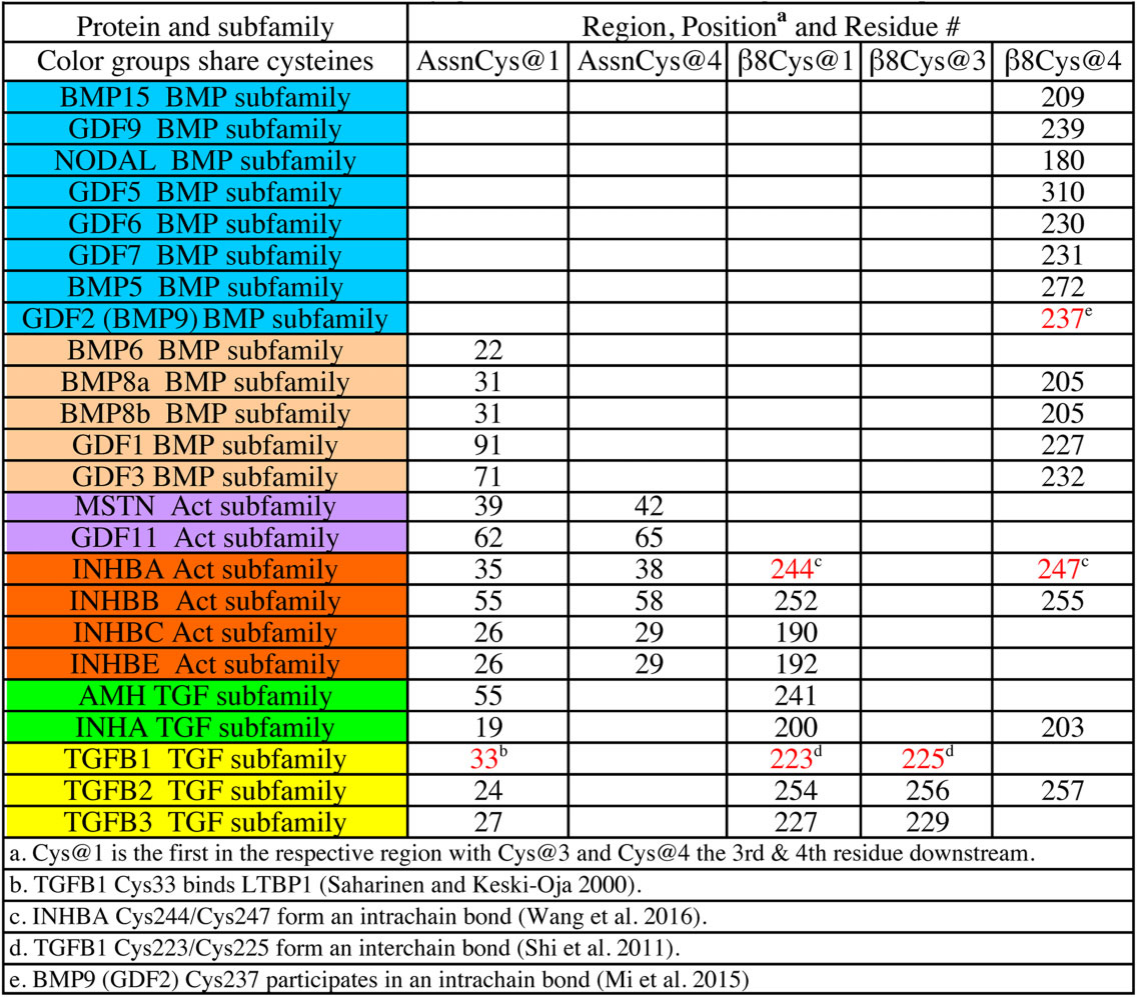

**Fig. 3.**
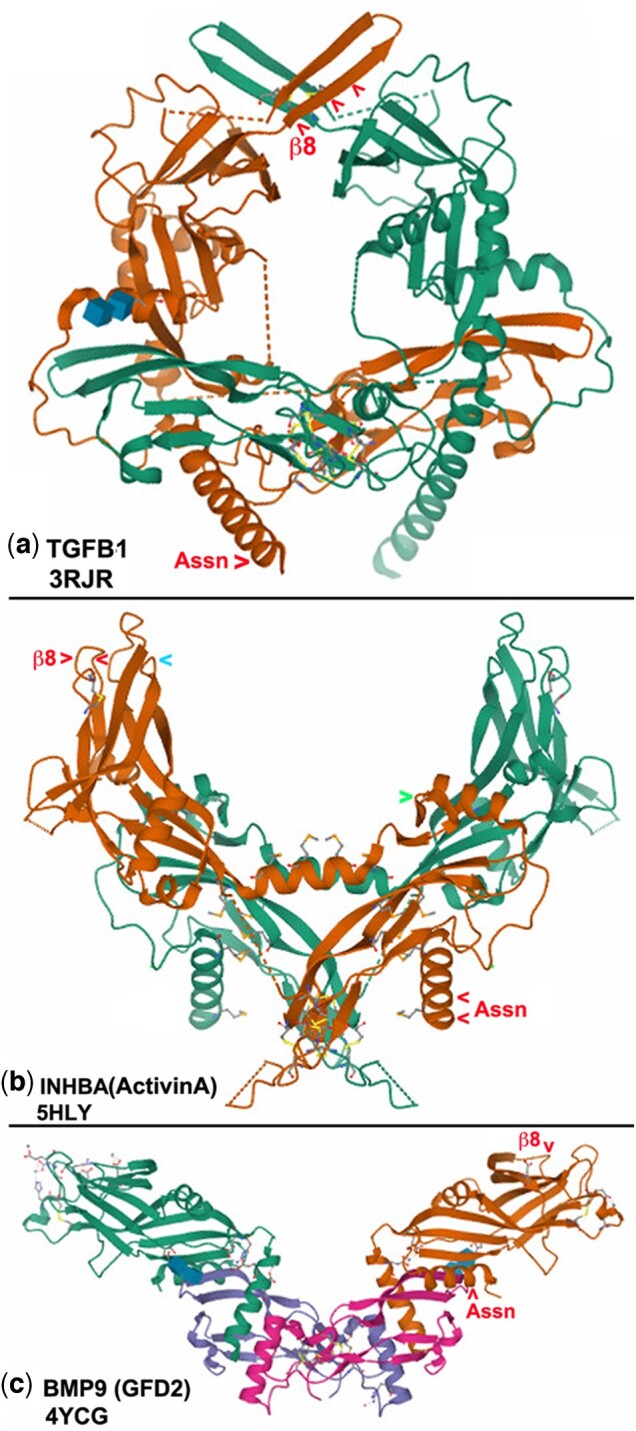
Conserved prodomain cysteines in 2 exposed structural regions. a) TGFB1. Crystal structure of the porcine prodomain dimer with monomers in green and brown. Red arrowheads indicate 4 conserved cysteines in the brown monomer seen in various combinations in 5 of 8 TGF-β subfamily members. The Assn region has 1 cysteine exposed near the amino terminus. The β8 region has 3 cysteines exposed near the carboxy-terminal cleavage site. The dimer has a closed-ring structure. Image created in Mol* (Sehnal *et al*. 2021; Burley *et al*. 2021) (www.rcsb.org/3d-view/3RJR). b) INHBA (ActivinA). Crystal structure of the human prodomain dimer with monomers in green and brown. Red arrowheads indicate 4 conserved cysteines in the brown monomer seen in various combinations in 6 of 8 Activin subfamily members. The Assn and β8 regions each have 2 cysteines in exposed locations. The blue arrowhead indicates IHNBB Arg223Cys a gain-of-function mutation found in endometrial tumors. The green arrowhead indicates IHNBB Ser154Cys a gain of function mutation found in plasma cell tumors. The dimer has an open-arm conformation (www.rcsb.org/3d-view/5HLY). c) BMP9 (GDF2). Crystal structure of the mouse complete dimer with the prodomains in green and brown. Red arrowheads indicate 2 conserved cysteines in the brown monomer seen in various combinations in 13 of 17 BMP subfamily members. The Assn and β8 regions each have 1 cysteine in exposed locations. The dimer has a widely open conformation (www.rcsb.org/3d-view/4YCG).

The conserved cysteine positions were then visualized in the 3 TGF-β subfamily prodomain structures (Fig. 3). The number of cysteines shown for each subfamily is a compilation and not every member has all of them. In all 3 structures, conserved cysteines are located in 2 highly exposed locations. The β8 region near the ligand cleavage site is part of a protruding loop in all subfamilies. The loop is composed of 2 β-sheets (β8 and β9) in the crystal structure of TGFB1. The conserved cysteine loop in the other 2 subfamilies does not contain β-sheets but still sits at the surface. In the β8 region, partners could be other TGF-β family members to form heterodimers. The Assn region near the signal sequence cleavage site also extends beyond the core of the structure in all subfamilies. The exposed locations suggest that their conserved cysteines participate in bonds with external partners. In the Assn region, partners could be one of 10 potential binding proteins in 4 families: LTBP, Fibrillin (FBN), Leucine Repeat Containing (LRC) or E-Selectin (SELE).

### Conserved cysteines in the β8 region of 21 human TGF-β proteins

We observed that all cysteine positions in the β8 region correspond to demonstrated functional cysteines (Table 1). One pair (TGFB1 Cys223/225; Shi *et al.* 2011) that we refer to β8Cys@1 and β8Cys@3 forms an interchain bond with their counterparts in a homodimer. A second functional cysteine pair (INHBA Cys244/247; Wang *et al.* 2016) that we refer to β8Cys@1 and β8Cys@4 forms an intrachain bond in a homodimer.

TGFB2, INHA, INHBA and INHBB also share cysteines β8Cys@1 and β8Cys@4. TGFB2 and INHA were predicted to form heterodimers in our prior study (Wisotzkey and Newfeld 2020) and both of these are known to heterodimerize with their siblings (Cheifetz *et al.* 1988; Walton *et al.* 2009). All other proteins with β8@Cys4 are BMP subfamily members. The structure of BMP9 homodimers showed that β8@Cys4 forms an intrachain bond (Cys237 in GDF2; synonym for BMP9).

Overall, there are 13 BMP, 4 Activin, and 4 TGF-β subfamily members with at least 1 conserved cysteine in β8. All conserved cysteine positions in the β8 region correspond to cysteines that participate in disulfide bonds in their respective subfamily structures. The discovery of cysteine conservation in the β8 region of 21 TGF-β proteins broadens the potential impact of disulfide-dependent regulation by heterodimerization from 10% to 64% of proteins.

### Conserved cysteines in the Assn region of 16 human TGF-β proteins

There are more conserved cysteine positions in the Assn region than known functional cysteines. The Assn region has 2 conserved cysteine positions (Table 1), but the only known functional cysteine position is AssnCys@1 (TGFB1 Cys33). Each monomer of a TGFB1 homodimer forms a disulfide linkage via this cysteine to either LTBP1 Cys1359 or Cys1384. TGFB1 also employs Cys33 to bind to LTBP3 and LTBP4. Conserved cysteines in this position in TGFB2 (Cys24) and TGFB3 (Cys27) are presumed to serve the same function, though this has not formally been shown. The fact that 16 proteins have an AssnCys@1 suggests that partner protein disulfide regulation extends to members of all 3 subfamilies.

The second Assn region-conserved cysteine (AssnCys@4) is present only in 6 Activin subfamily proteins. These are the 4 INHBs plus MSTN and GDF11. The latter 2 are the only proteins with 2 Assn region cysteines and no β8 region cysteines. With the 2 Assn cysteines showing identical spacing to β8Cys@1 and β8Cys@4 and the Assn region also highly exposed, 1 new hypothesis is that the 2 Assn region cysteines facilitate an Activin subfamily-specific mechanism for heterodimerization.

Overall, there are 5 BMP, 6 Activin and 5 TGF-β subfamily members with at least 1 conserved cysteine in this region. All 16 sequences contain AssnCys@1 with 6 also displaying AssnCys@4. The highly exposed nature of the Assn region together with the role of TGFB1 AssnCys@1 in binding LTBP1/LRC32 suggests that a disulfide-dependent binding partner regulates as many as 48% of human TGF-β proteins.

### Common phenotype of prodomain region cysteine mutations indicates new heterodimers

To assign specific interactions to individual cysteines, we mined 3 databases of disease-associated mutations. We applied the concept originated by Beadle and Tatum (1941) for the dissection of biochemical pathways: common mutant phenotypes result from a lost biochemical interaction that would normally achieve a common function.

The phenotypes of 9 conserved prodomain cysteines that display a loss-of-function mutation are shown (Table 2). Common phenotypes in different proteins would broaden the impact of regulation by heterodimerization in the TGF-β family. In the table, mutations are color-coded and numbered to display potential within subfamily and across subfamily heterodimer pairs. Eight cysteine mutations are in the β8 region and 1 is in the Assn region.

**Table 2.** Common disease phenotypes of cysteine mutations suggest 3 new across subfamily heterodimers and 4 new within subfamily heterodimers.

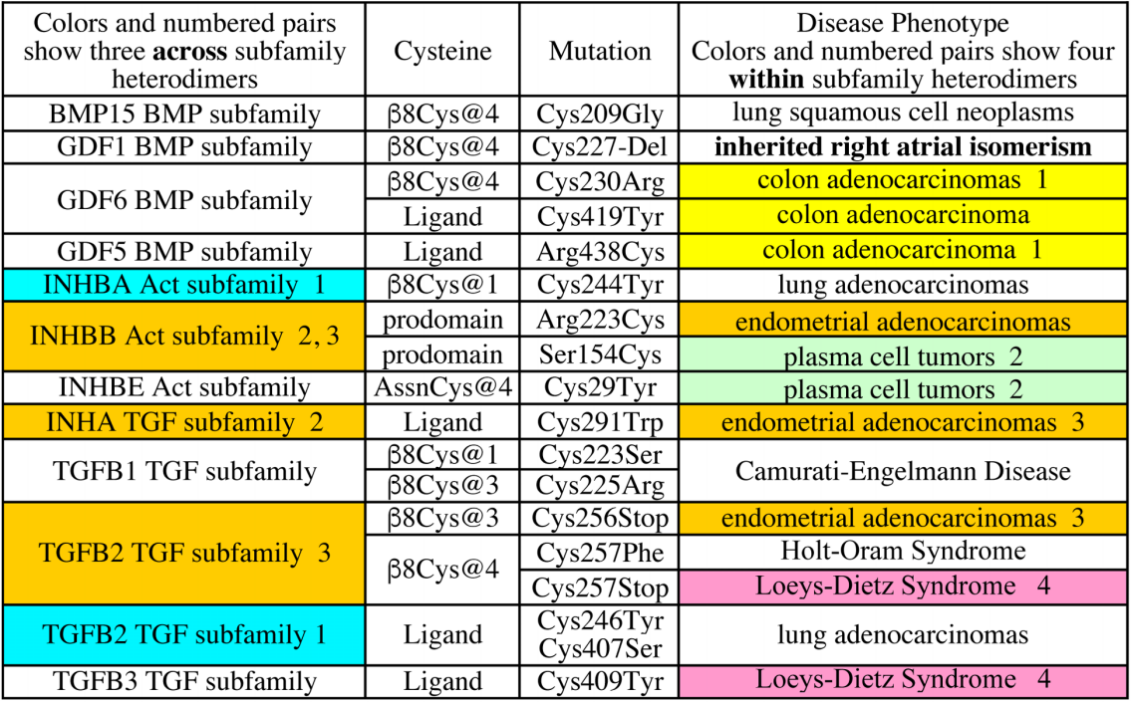

There are 3 mutations in BMP subfamily members. BMP15 β8Cys@4 has Cys209Gly found in lung squamous cell neoplasms. GDF1 β8Cys@4 Cys227-Del is an in-frame deletion of 145 residues causing inherited right atrial isomerism, a heart defect. For GDF6, there are 2 colon adenocarcinoma mutations, 1 in β8Cys4 (Cys230Arg) and 1 in the ligand (Cys419Tyr). Furthermore, a GDF5 ligand mutation Arg438Cys is also associated with colon adenocarcinomas. When a ligand cysteine mutation generates the same phenotype as a conserved prodomain cysteine mutation, it provides additional confidence that the prodomain cysteine is essential. The common phenotype of GDF5 and GDF6 suggests heterodimerization.

Six mutations are in Activin and TGF-β subfamily members. INHBA β8Cys@1 has Cys244Tyr found in lung adenocarcinomas. Note that BMP15 and INHBA cancers originate in distinct cell types (squamous cells vs epithelial cells, respectively), limiting the relevance of these mutations to understand heterodimerization. TGFB1 β8Cys@1 has 3 mutations Cys223Arg/Gly/Ser and TGFB1 β8Cys@3 has 2 Cys225Arg/Tyr. All 5 can cause Camurati-Engelmann disease, an autosomal dominant disease with skeletal hyperplasia.

INHBE AssnCys@4 has Cys29Tyr that is associated with plasma cell tumors. INHBB has a prodomain gain-of-function mutation that is also associated with plasma cell tumors. IHNBB Ser154Cys maps to an exposed loop between β1 and β2 that is topologically near to the Assn region in the INHBA structure (Fig. 3b, green arrowhead right). A new cysteine in close proximity to the INHBB pair of Assn cysteines likely interferes with their function. If INHBE and INHBB formed a heterodimer or were regulated by a common binding partner, then loss or gain of cysteine near the Assn region could be disruptive and result in tumors.

TGFB2 β8Cys@3 has Cys256Stop identified in endometrial adenocarcinomas. The same tissue and tumor type are seen with the Cys291Trp in the ligand of INHA that shares β8Cys@1 and β8Cys@4 with TGFB2. Endometrial adenomas were again seen with Arg223Cys in the prodomain of INHBB that shares β8Cys@1 and β8Cys@4 with TGFB2 and INHA. Arg223Cys maps to an exposed loop between β6 and β7 that is topologically near to β8 in the INHBA structure (Fig. 3b, blue arrowhead left). A new cysteine in close proximity to the pair of INHBB β8 region cysteines likely interferes with their function. The common phenotype for INHA and INHBB that is known to heterodimerize increases confidence in identifying heterodimer partners via cysteine mutations and common disease phenotypes. The fact that TGFB2 shares 2 β8 cysteines and an endometrial adenoma phenotype with INHA and INHBB implies that TGFB2 can heterodimerize with either to prevent endometrial tumor formation.

TGFB2 β8Cys@4 has 2 mutations. First, Cys257Phe is associated with Holt–Oram syndrome. This is an autosomal dominant disease with a proximate cause of nonfunctional TBX5 (Boogerd *et al*. 2010). It is characterized by skeletal abnormalities and heart defects. A TGFB2 ligand mutation Cys378Tyr is also found in Holt-Oram.

The TGFB2 mutation Cys257Stop in β8Cys@4 was noted in Loeys–Dietz syndrome. This is an autosomal dominant disease with systemic effects on connective tissue and blood vessels. A TGFB2 ligand mutation Cys439Ser was also noted in Loeys–Dietz, as was a ligand mutation in TGFB3 Cys409Tyr. The common phenotype for these mutations suggests heterodimerization. Demonstrated heterodimers of TGFB2 and TGFB3 (Cheifetz *et al.* 1988) serve to validate this hypothesis. What is new is that TGFB2 β8Cys4 shows the phenotype, thus suggesting a β8-based mechanism for TGFB2 and TGFB3 heterodimer formation.

Cysteine mutations in TGFB2, INHA, and INHBB associated with endometrial adenocarcinoma plus cysteine mutations in TGFB2 and TGFB3 with Loeys–Dietz syndrome lead to the hypothesis that TGFB2 is capable of heterodimerization with numerous partners. Further evidence for TGFB2 versatility is that 2 TGFB2 ligand mutations Cys246Tyr and Cys407Ser share a lung adenocarcinoma phenotype with a mutation in β8Cys@1 of INHBA Cys244Tyr. Taken together, TGFB2 cysteine mutations have a common phenotype with cysteine mutations in 4 TGF-β family members INHA, INHBA, INHBB, and TGFB3.

Overall, phenotypic analyses of 9 prodomain-conserved cysteine disease mutations in 7 proteins suggested 7 new heterodimers. Four heterodimers are within a subfamily (1 BMP, 1 Activin, and 2 TGF-β) and 3 are across subfamilies (all are Activin with TGF-β). Six heterodimers are associated with tumors, with 5 of these adenomas. We then identified common disease phenotypes shared by conserved prodomain cysteine mutations and partner-binding protein cysteine mutations.

### Common phenotype of Assn region cysteine mutations suggest new regulation

We analyzed the single Assn region mutation separately from the β8 region mutations. A common phenotype for the Assn region cysteine mutation and a cysteine mutation in one of the 10 potential TGF-β-binding proteins would suggest a regulatory interaction that broadens the impact of disulfide-dependent partner protein binding. INHBE Cys29Tyr in AssnCys@4 is found in plasma cell tumors from the hematopoietic and phagocytic systems. This suggests that undetected binding partners can regulate Activin subfamily members.

We identified all cysteine mutations showing a plasma cell tumor phenotype in each potential binding partner (LTBP1–4, FBN1–3, LRC32/33, and SELE). We found 11 cysteine mutations in 4 proteins that generate this phenotype (Table 3). There is 1 plasma cell tumor cysteine mutation in LTBP1. Cys1022Tyr is in EGF-like repeat-7, roughly 330 residues upstream of TGF-β-binding domain-3 that binds TGFB1. The common phenotype with INHBE suggests an interaction between LTBP1 and INHBE via AssnCys@4. The location of LTBP1 Cys1022 outside of TGF-β-binding domain-3 is logical since INHBA has its Assn cysteines in a topologically distinct location from TGFB1 (Fig. 3b).

**Table 3.** Common disease phenotype for an INHBE Assn region cysteine mutation suggests 4 new binding partner interactions associated with plasma cell tumors.

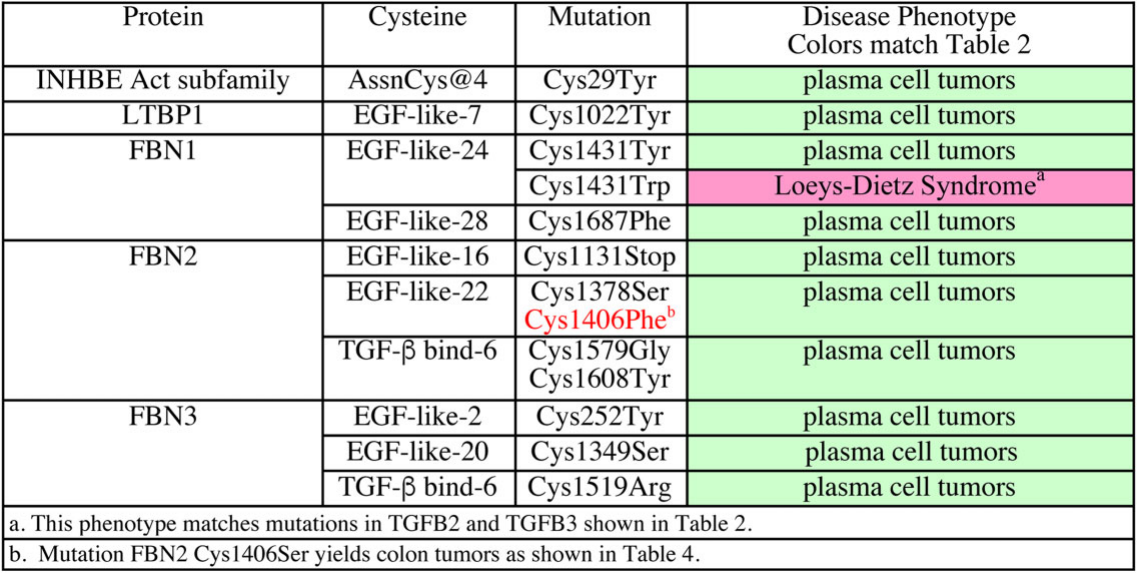

There are 2 plasma cell tumor cysteine mutations in FBN1. One is Cys1431Tyr in EGF-like repeat-24 and the other Cys1687Phe is in EGF-like repeat-28. The common phenotype suggests an interaction between FBN1 and INHBE via AssnCys@4. Another FBN1 mutation Cys1431Trp is associated with Loeys–Dietz syndrome, a phenotype generated by prodomain cysteine mutations in TGFB2 and TGFB3. The common Loeys–Dietz syndrome phenotype for cysteine mutations in FBN1, TGFB2, and TGFB3 supports their interaction via conserved prodomain cysteines.

**Fig. 4.**
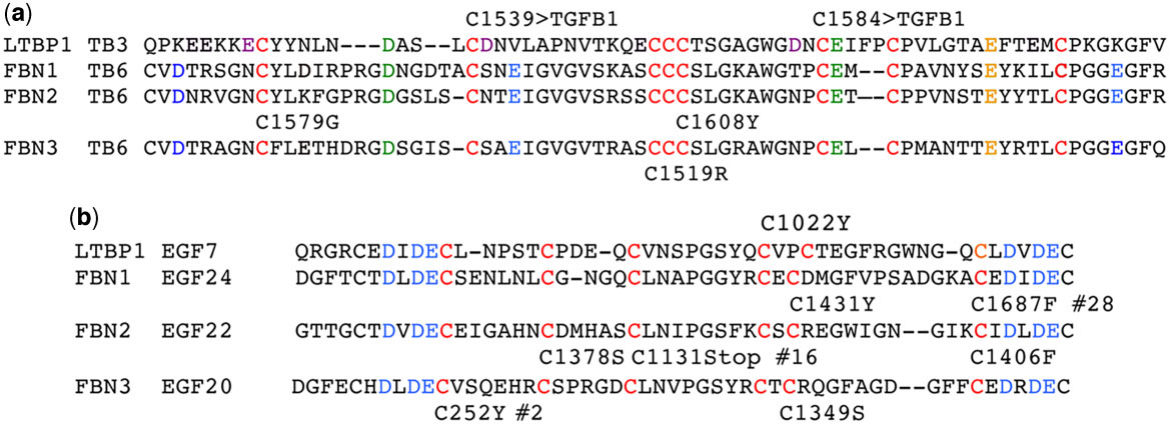
Conserved cysteine mutations and acidic residues are aligned in LTBP1 and FBN TGF-β binding and EGF-like domains. a) Top row is the LTBP1 TGF-β-binding domain-3 (TB3) domain with its 8 cysteines in red. The 2 cysteines that bind TGFB1 are indicated above the sequence. The sequence of TGF-β-binding domain-6 (TB6) in each of the 3 FBN family members is aligned. Three cysteine mutations in FBN2 and FBN3 that are found in plasma cell tumors are shown below their sequence. Note the expected gap in FBN proteins opposite the LTBP1 FP insertion that is unique to TGFB1 binding. Acidic residues (D/E) in the docking site for TGFB1 are in green (D12 and E42) if they are conserved in FBN sequences and in purple (E5, D17, and D39) if they are not. Three D/E residues present in all FBN sequences but that are absent in LTBP1 are in blue. An additional conserved E in all 4 sequences with no known function is in orange. b) Top row is the LTBP1 EGF-like repeat-7 with its 6 cysteines in red. One EGF-like repeat with a cysteine mutation from each FBN family member is aligned. A cysteine mutation in LTBP1 and 7 in FBN proteins that are associated with plasma cell tumors are indicated. Three cysteine mutations present in other FBN EGF-like repeats are indicated by their repeat numbers (#28, #16, and #2). Five of the 6 cysteines in a canonical EGF-like repeat are mutated in plasma cell tumors. Three D/E residues immediately upstream of the first cysteine of the repeat and 3 D/E residues immediately downstream of the last are in blue. All 6 are present in LTBP1 and the 3 FBN sequences.

There are 5 plasma cell tumor cysteine mutations in FBN2. One is in EGF-like repeat-16 and 2 are in EGF-like repeat-22 including Cys1406. Mutations in Cys1406 have distinct associations: Cys1406Phe is found in plasma cell tumors and Cys1406Ser is associated with colon adenocarcinoma. The mutations Cys1579Gly and Cys1608Tyr are in TGF-β-binding domain-6. Their spacing of 27 amino acids is similar to the spacing of the TGFB1-binding cysteines in LTBP1 (25 residues). An alignment of FBN2 TGF-β-binding domain-6 and LTBP1 TGF-β-binding domain-3 (Fig. 4a) shows that both FBN2 mutant cysteines are spaced similarly to those in LTBP1. Spacing similarity supports the hypothesis that these cysteines in FBN2 TGF-β-binding domain-6 are each capable of binding a monomer of INHBE AssnCys@4.

There are 3 plasma cell tumor cysteine mutations in FBN3. They are in EGF-like repeat-2, EGF-like repeat-20 and in TGF-β binding domain-6. An alignment of TGF-β-binding domain-6 from FBN3 with TGF-β-binding domain-6 in FBN2 (Fig. 4a) shows that Cys1519Arg impacts the same cluster of 3 cysteines as FBN2 Cys1608Tyr mutation and yields the same phenotype. Phenotypic correspondence provides additional evidence in support of the hypothesis that FBN family members bind to Activin subfamily Assn region cysteines.

Independent support for the FBN-Activin-binding hypothesis derives from an NMR study of FBN1 TGF-β-binding domain-6 and the adjacent downstream EGF-like repeat (Yuan *et al.* 2002). The Marfan syndrome mutation N2144S in the FBN1 EGF-like repeat led to a conformational shift impacting the 2 nearest intrachain disulfide bonds. The loss of those bonds altered the protein's local topology in both directions including TGF-β-binding domain-6. The authors conclude that lost interactions of TGF-β-binding domain-6 contribute to the disease.

Overall, the common phenotype analysis of an Assn region cysteine mutation in INHBE suggested 4 new binding partners. Loss of each regulatory interaction results in plasma cell tumors suggesting that regulation serves to prevent an IHNBE promitotic signal from impacting the hematopoietic and/or phagocytic systems.

### Common phenotype of β8 region cysteine mutations suggests additional new regulation

Analysis of β8 region cysteines began with GDF6 β8Cys@4 (Cys230Arg) that is found in colon adenocarcinomas (Table 4). Five partner proteins display cysteine mutations found in this tumor. In LTBP1, 2 plasma cell mutations in TGF-β-binding domain-1 Cys559Tyr and Cys594Trp are 35 residues apart affecting the first and sixth cysteines. In LTBP1 TGF-β-binding domain-3, the cysteines that bind TGFB1 are the second and sixth. Perhaps the extra space is necessary to accommodate the widely open structure of BMP proteins. Each of the 3 FBN proteins has at least 3 cysteine mutations linked to colon adenocarcinomas. In FBN3, Cys1406Ser occurs in the same cysteine where Cys1406Phe was found in plasma cell tumors. Distinct missense mutations in a single FBN3 cysteine in tumors linked to BMP and Activin proteins indicate that FBN3 may have multiple partners.

**Table 4.** Common disease phenotypes for GDF6 and TGFB2 β8 region cysteine mutations suggest 13 new binding partner interactions associated with tumors.

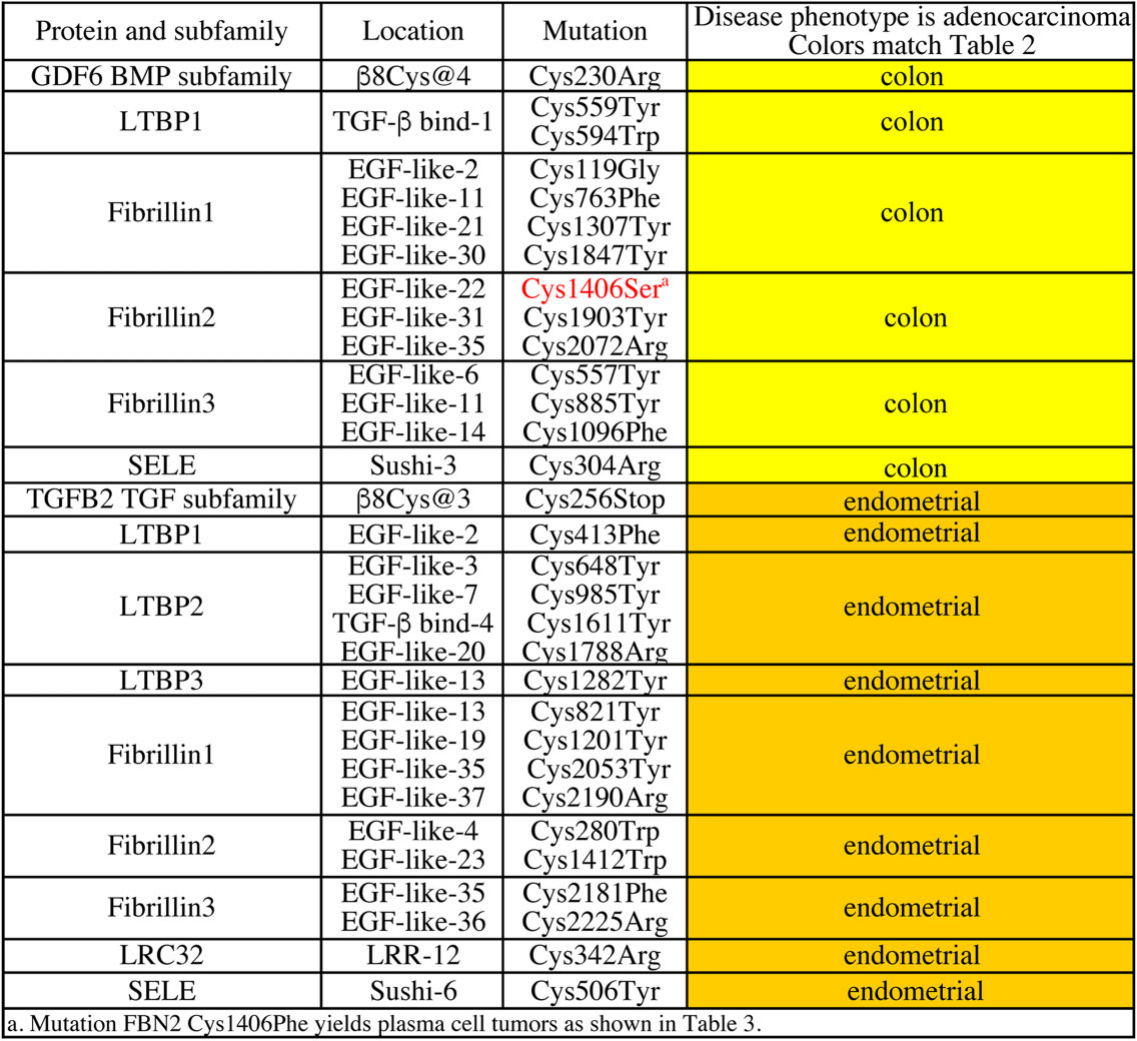

A second β8 region mutation TGFB2 β8Cys@3 (Cys256Stop) is found in endometrial adenocarcinomas. Eight partner proteins display cysteine mutations in this tumor. Four of these have a single mutation LTBP1, LTBP3, LRC32, and SELE. The Cys342Arg mutation in LRC32 is in the same repeat (LRR-12) as Cys350 that binds to TGFB1. These are the only 2 cysteines in this repeat. There are 2 mutant cysteines each in FBN2 and FBN3 and 4 mutant cysteines in LTBP2 and FBN1.

Overall, the common phenotype analysis of β8 region cysteine mutations in GFD6 and TGFB2 suggested 13 new binding partners. Loss of each regulatory interaction results in adenocarcinomas suggesting that regulation serves to prevent a GDF6 or TGFB2 promitotic signal from impacting the colon or endometrium, respectively.

Taken together, the common phenotype analysis of Assn and β8 region-conserved cysteine mutations (1 protein from each subfamily) suggested 17 new regulatory interactions with 8 binding partners. Sixteen of these interactions are associated with tumors. Four of the partners (LTBP1 and FBN1-3) demonstrate a common disease phenotype with each mutant TGF-β protein suggesting the ability to bind to members of all 3 subfamilies. As all regulatory partners act to restrict signaling, common mutant phenotypes in tumors suggest that the absence of partner binding (due to a prodomain or a partner cysteine mutation) contributes to TGF-β family members’ well-known ability to serve tumors as promitotic signals.

### LTBP1 repeat that binds TGFB1 aligns well with repeats in FBN proteins

To investigate the basis for the common phenotypes noted above for FBN1-3 cysteine mutations, we created alignments with LTBP1 of FBN1-3 TGF-β-binding domain-6 (Fig. 4a) and an EGF-like repeat (Fig. 4b). Two important features of TGF-β-binding domain-3 in LTBP1 that shape its ability to bind TGFB1 Cys33 are: (1) a 2 residue (FP) insertion between cysteines 6 and 7 and (2) a suite of 5 acidic residues (D/E) that form a docking site (Lack *et al.* 2003; Chen *et al.* 2005). The fact that FBN proteins do not bind TGFB1 is attributed to the lack of the FP insertion in any of their TGF-β-binding domains (Rifkin *et al.* 2022). Data in Table 3 address the question “would the absence of the insertion prevent Activin subfamily binding?” The common plasma cell tumor phenotype for a cysteine mutation in INHBE and 3 mutations in TGF-β-binding domain-6 of FBN2 and FBN3 suggest it does not.

Similarly, a different 5 D/E residue docking site would be expected for Activin subfamily binding by an FBN TGF-β-binding domain. Different docking sites are needed to accommodate the cross-arm structure of Activin proteins vs the closed-ring of TGF-β proteins. A comparison of FBN TGF-β-binding domain-6 D/E content to LTBP1 TGF-β-binding domain-3 reveals that 2 of the D/E residues that form 1 edge of the docking site in LTBP1 TGF-β-binding domain-3 are conserved in all 3 FBN sequences (D12, E42; Chen *et al.* 2005). Three other D/E residues are not (E5, D17, D39). Instead there are 3 D/E residues aligned in all FBN sequences that are spaced similarly. The 5 D/E docking sites in FBN TGF-β-binding domain-6 with a slightly different shape for Activin proteins seem likely.

Overall, the mutant phenotype analysis expands the scope of TGF-β family partner interactions to include EGF-like repeats in LTBP1 and the 3 FBN proteins. Eight cysteine mutations in EGF-like repeats in LTBP1 and in the 3 FBN proteins are associated with tumors. Two cysteine mutations in EGF-like repeat-22 of FBN2 show spacing of 28 residues similar to the spacing of LTBP1 TGFB1-binding cysteines. Finally, 6 acidic resides are present in EGF-like repeats of LTBP1 and the FBN proteins that could form a docking site for an Activin protein.

## Discussion

Frequent regulatory interactions with partner proteins seem likely given the number of cysteines in each potential partner protein. The secreted LTBP and FBN families each contain multiple 8-Cys repeat domains. An 8-Cys repeat in LTBP1 contains the 2 cysteines that each bind to Cys33 in a monomer of TGFB1 (Saharinen and Keski-Oja 2000). As a result, 8-Cys repeats in the 4 LTBP and 3 FBN proteins are also called TGF-β-binding domains. Proteins in these 2 families also contain a large number of 6-Cys EGF-like repeats. For example, LTBP1 has 16 and FBN1 has 47 of them. While EGF-like repeats are common, 8-Cys repeats are not found in any other protein. To date no TGF-β partner for LTBP2 is known and only TGFB1–3 have been tested for binding to LTBP1–4.

Studies of FBN1 binding to TGFB1 have been negative, dampening enthusiasm for this family (Yuan *et al.* 2002). As a result, to our knowledge, no other TGF-β family members have been tested for binding to any FBN protein. Each FBN protein has 8 TGF-β-binding domains. Alignments indicate that TGF-β-binding domains in FBN family members share features with TGF-β-binding domain-3 in LTBP1 that binds TGFB1 Cys33. There are 105 potential partnerships between the 15 TGF-β proteins that are not TGFB1 but have AssnCys@1 and the 7 TGF-β-binding domain proteins (4 LTBP plus 3 FBN proteins). The probability that a subset of these interactions is real seems high.

The cell surface transmembrane proteins LRC32 (also known as GARP) and LRC33 are also TGFB1-binding partners. The extracellular portion of LRC32 binds to TGFB1 Cys33 in the same 2:1 ratio as LTBP1 (Liénart *et al.* 2018). In contrast to the proximity of the 2 cysteines in LTBP1 (Cys1539/1584, 25 residues), the 2 cysteines in LRC32 are far apart (Cys211/350, 139 residues). TGFB1 binding to LRC33 has been reported but the relevant cysteines are not identified (Ma *et al.* 2019). These proteins have roughly 20 Leucine Rich Repeats containing 1 or 2 cysteines each. It again seems likely that additional TGF-β proteins can bind LRC32 and LRC33.

The ER transmembrane protein E-Selectin-ligand (SELE) plays a different role. Rather than maintaining latency in the secreted TGFB1 dimer like the others, this binding partner is a negative regulator of TGFB1 and TGFB2 secretion (Yang *et al.* 2010). Neither the cysteine in SELE nor the cysteine in TGFB1 and TGFB2 is known. SELE contains 6 Sushi domains each with 6 cysteines. The potential for a disulfide bond between SELE and the 14 TGF-β family proteins with AssnCys@1 that are not TGFB1 and TGFB2 is considerable.

Nevertheless, we recognize that not all the tumor phenotypes provided by the NCI Cancer Genome Commons precisely describe a specific cell type. While colon, endometrial, and lung adenocarcinoma describe only epithelial cell tumors, the phenotype plasma cell tumors covers many cell types. We also recognize that heterodimerization and partner binding require expression of both proteins in the same cell. To date, for most human TGF-β family proteins, detailed expression data to confirm or deny our hypotheses are lacking. Thus, we urge our colleagues to test our hypothesis for new heterodimers and new binding partner interactions.

In conclusion, for over 20 years, our understanding of TGF-β signaling has benefited from computational approaches. A recent advance in our ability to align TGF-β family prodomains across species facilitated this study of human TGF-β prodomain cysteines. New human prodomain alignments revealed that 24 TGF-β prodomains contain conserved cysteines in 2 highly exposed locations. There are 3 in the region of the β8 helix that mediates dimerization near the prodomain carboxy terminus. There are 2 in the Assn region that mediates partner protein binding near the prodomain amino terminus. The alignments predict the specific cysteines contributing to disulfide-dependent regulation of 72% of human TGF-β proteins. Database mining then identified 9 conserved prodomain cysteine mutations and their disease phenotypes in 7 TGF-β proteins. Three common adenoma phenotypes for prodomain cysteine mutations suggested 7 new regulatory heterodimer pairs. Two common adenoma phenotypes for prodomain and binding partner cysteine mutations revealed 17 new regulatory interactions. Overall, the analysis of human TGF-β prodomains suggests a significantly expanded scope of disulfide-dependent regulation by heterodimerization and partner protein binding; regulation that is often lost in tumors.

## Supplementary Material

jkac271_Supplementary_Data

## Data Availability

The authors affirm that all data necessary for confirming the conclusions of the article are present in the article, figures, tables, and supplemental information. Supplemental material is available at G3 online.
