# Supplementary material for: Computational analysis of prodomain cysteines in human TGF-β proteins reveals frequent loss of disulfide-dependent regulation in tumors
Source: G3 (Bethesda). 2022 Oct 10;12(12):jkac271. doi: 10.1093/g3journal/jkac271 (PMC9713452; doi:10.1093/g3journal/jkac271)
Supplement: jkac271_Supplementary_Data [file jkac271_supplementary_data.pdf]

Supplemental Material: Two Tables

Table S1. Human TGF-β superfamily sequences, subfamilies and accession numbers.

Subfamily	Name	Accession	Description (synonym)
Activin	BMP3	NP_001192.4	bone morphogenetic protein 3 preproprotein
Activin	GDF10	NP_004953.1	growth/differentiation factor 10 preproprotein
Activin	GDF11	NP_005802.1	growth/differentiation factor 11 preproprotein (syn. BMP11)
Activin	INHBA	NP_002183.1	inhibin beta A chain preproprotein
Activin	INHBB	NP_002184.2	inhibin beta B chain preproprotein
Activin	INHBC	NP_005529.1	inhibin beta C chain preproprotein
Activin	INHBE	NP_113667.1	inhibin beta E chain preproprotein
Activin	MSTN	NP_005250.1	growth/differentiation factor 8 preproprotein (synonym GDF8)
8 Activin			
BMP	BMP2	NP_001191.1	bone morphogenetic protein 2 preproprotein
BMP	BMP4	NP_001193.2	bone morphogenetic protein 4 isoform a preproprotein
BMP	BMP5	NP_066551.1	bone morphogenetic protein 5 isoform 1 preproprotein
BMP	BMP6	NP_001709.1	bone morphogenetic protein 6 preproprotein
BMP	BMP7	NP_001710.1	bone morphogenetic protein 7 preproprotein
BMP	BMP8a	NP_861525.2	bone morphogenetic protein 8A preproprotein
BMP	BMP8b	AAP74560.1	bone morphogenetic protein 8B
BMP	BMP10	NP_055297.1	bone morphogenetic protein 10 preproprotein
BMP	BMP15	NP_005439.2	bone morphogenetic protein 15 preproprotein (synonym GDF9b)
BMP	GDF1	NP_001483.3	embryonic growth/differentiation factor 1 precursor
BMP	GDF2	NP_057288.1	growth/differentiation factor 2 preproprotein (synonym BMP9)
BMP	GDF3	NP_065685.1	growth/differentiation factor 3 preproprotein
BMP	GDF5	NP_000548.2	growth / differentiation factor 5 preproprotein
BMP	GDF6	NP_001001557.1	growth/differentiation factor 6 preproprotein
BMP	GDF7	NP_878248.2	growth/differentiation factor 7 preproprotein
BMP	GDF9	NP_ 005251.1	growth/differentiation factor 9 preproprotein
BMP	NODAL	NP_060525.3	nodal homolog isoform 1 preproprotein
17 BMP			
TGF-β	AMH	NP_000470.3	mullerian inhibiting factor preproprotein (synonym MIS)
TGF-β	GDF15	NP_004855.2	growth/differentiation factor 15 preproprotein
TGF-β	INHA	NP_002182.1	inhibin alpha chain isoform 1preproprotein
TGF-β	LEFTY1	NP_066277.1	left-right determination factor 1 preproprotein
TGF-β	LEFTY2	NP_003231.2	left-right determination factor 2 isoform 1 preproprotein
TGF-β	TGFB1	NP_000651.3	transforming growth factor beta-1 preproprotein
TGF-β	TGFB2	NP_001129071.1	transforming growth factor beta-2 isoform 1 precursor
TGF-β	TGFB3	NP_001316868.1	transforming growth factor beta-3 isoform 1 preproprotein
8 TGF-β			
33 total TGF-β superfamily sequences			
Outgroup	GDNF	NP_001177397.1	glial cell line-derived neurotrophic factor isoform 3

Table S2. Human TGF-β superfamily signal sequences and ligand cleavage sites.

Subfamily	Name	Signal Sequence Residues[a]	Prodomain side	Cleavage Site[b]	Ligand side
Activin	BMP3	1 - 22	ALSIER**RKKR**	//	STGVLLPLQN
Activin	GDF10	1 - 23	LKPRPG**RKDR**	//	RKKGQEVFMA
Activin	GDF11	1 - 24	VLENTK**RSRR**	//	NLGLDCDEHS
Activin	INHBA	1 - 20	SEDHPH**RRRR**	//	RGLECDGKVN
Activin	INHBB	1 - 28	RLGDSR**HRIR**	//	KRGLECDGRT
Activin	INHBC	1 - 18	RVGGKH**QIHR**	//	RGIDCQGGSR
Activin	INHBE	1 - 19	EPGAGR**ARRR**	//	TPTCEPETPL
Activin	MSTN	1 - 23	VTDTPK**RSRR**	//	DFGLDCDEHS
BMP	BMP2	1 - 23	GHPLHK**REKR**	//	QAKHKQRKRL
BMP	BMP4	1 - 19	ALTRRR**RAKR**	//	SPKHHSQRAR
BMP	BMP5	1 - 30	ASEVLL**RSVR**	//	AANKRKNQNR
BMP	BMP6	1 - 20	VSEVHV**RTTR**	//	SASSRRRQQS
BMP	BMP7	1 - 29	ATEVHF**RSIR**	//	STGSKQRSQN
BMP	BMP8a	1 - 19	ASPSPI**RTPR**	//	AVRPLRRRQP
BMP	BMP8b	1 - 19	SPIRTP**RAVR**	//	PLRRRQPKKS
BMP	BMP10	1 - 21	IYDSTA**RIRR**	//	NAKGNYCKRT
BMP	BMP15	1 - 18	ERESLL**RRTR**	//	QADGISAEVT
BMP	GDF1	1 - 29	GPGGAC**RARR**	//	DAEPVLGGGP
BMP	GDF2	1 - 22	AGSTLA**RRKR**	//	SAGAGSHCQK
BMP	GDF3	1 - 24	DQCHPS**RKRR**	//	AAIPVPKLSC
BMP	GDF5	1 - 27	EYLFSQ**RRKR**	//	RAPLATRQGK
BMP	GDF6	1 - 22	KRHGKK**SRLR**	//	CSKKPLHVNF
BMP	GDF7	1 - 19	AGRGHG**RRGR**	//	SRCSRKPLHV
BMP	GDF9	1 - 24	GRSSHH**RHRR**	//	GQETVSSELK
BMP	NODAL	1 - 26	SWEWGK**RHRR**	//	HHLPDRSQLC
TGF-β	AMH	1 - 24	DPRGPG**RAQR**	//	SAGATAADGP
TGF-β	GDF15	1 - 29	LRPQAA**RGRR**	//	RARARNGDHC
TGF-β	INHA	1 - 18	PPSGGE**RARR**	//	STPLMSWPWS
TGF-β	LEFTY1	1 - 21	LASGAH**KLVR**	//	FASQGAPAGL
TGF-β	LEFTY2	1 - 21	LASGAH**KLVR**	//	FASQGAPAGL
TGF-β	TGFB1	1 - 29	QHLQSS**RHRR**	//	ALDTNYCFSS
TGF-β	TGFB2	1 - 20	SQQTNR**RKKR**	//	ALDAAYCFRN
TGF-β	TGFB3	1 - 23	PGQGGQ**RKKR**	//	ALDTNYCFRN
Outgroup	GDNF	1 - 20	QMAVLP**RRER**	//	NRQAAAANPE

a. Source: www.uniprot.org
b. Source: Wistozkey and Newfeld; Genetics 2020
